# Increasing Respiratory Effort With 100% Oxygen During Resuscitation of Preterm Rabbits at Birth

**DOI:** 10.3389/fped.2019.00427

**Published:** 2019-10-22

**Authors:** Janneke Dekker, Stuart B. Hooper, Michelle K. Croughan, Kelly J. Crossley, Megan J. Wallace, Erin V. McGillick, Philip L. J. DeKoninck, Marta Thio, Tessa Martherus, Gary Ruben, Charles C. Roehr, Sophie J. E. Cramer, Andreas W. Flemmer, Linda Croton, Arjan B. te Pas, Marcus J. Kitchen

**Affiliations:** ^1^Division of Neonatology, Department of Pediatrics, Leiden University Medical Center, Leiden, Netherlands; ^2^The Ritchie Centre, Hudson Institute of Medical Research, Melbourne, VIC, Australia; ^3^Department of Obstetrics and Gynaecology, Monash University, Melbourne, VIC, Australia; ^4^School of Physics and Astronomy, Monash University, Melbourne, VIC, Australia; ^5^Department of Obstetrics and Gynaecology, Erasmus MC, University Medical Center, Rotterdam, Netherlands; ^6^Women's Newborn Research Centre, The Royal Women's Hospital, Melbourne, VIC, Australia; ^7^Centre of Research Excellence in Newborn Medicine, Murdoch Children's Research Institute, Melbourne, VIC, Australia; ^8^Department of Obstetrics and Gynaecology, University of Melbourne, Melbourne, VIC, Australia; ^9^Newborn Services, John Radcliffe Hospital, Oxford University Hospitals, NHS Foundation Trust, Oxford, United Kingdom; ^10^Medical Sciences Division, Department of Pediatrics, University of Oxford, Oxford, United Kingdom; ^11^Department of Instrumental Affairs, Leiden University Medical Center, Leiden, Netherlands; ^12^Department of Neonatology, Dr. v. Haunersches Kinderspital & Perinatal Center Grosshadern, Medical Center of the University of Munich, Munich, Germany

**Keywords:** preterm, respiratory effort, oxygen, resuscitation, apnea, birth

## Abstract

**Background:** Spontaneous breathing is essential for successful non-invasive respiratory support delivered by a facemask at birth. As hypoxia is a potent inhibitor of spontaneous breathing, initiating respiratory support with a high fraction of inspired O_2_ may reduce the risk of hypoxia and increase respiratory effort at birth.

**Methods:** Preterm rabbit kittens (29 days gestation, term ~32 days) were delivered and randomized to receive continuous positive airway pressure with either 21% (*n* = 12) or 100% O_2_ (*n* = 8) via a facemask. If apnea occurred, intermittent positive pressure ventilation (iPPV) was applied with either 21% or 100% O_2_ in kittens who started in 21% O_2_, and remained at 100% O_2_ for kittens who started the experiment in 100% O_2_. Respiratory rate (breaths per minute, bpm) and variability in inter-breath interval (%) were measured from esophageal pressure recordings and functional residual capacity (FRC) was measured from synchrotron phase-contrast X-ray images.

**Results:** Initially, kittens receiving 21% O_2_ had a significantly lower respiratory rate and higher variability in inter-breath interval, indicating a less stable breathing pattern than kittens starting in 100% O_2_ [median (IQR) respiratory rate: 16 (4–28) vs. 38 (29–46) bpm, *p* = 0.001; variability in inter-breath interval: 33.3% (17.2–50.1%) vs. 27.5% (18.6–36.3%), *p* = 0.009]. Apnea that required iPPV, was more frequently observed in kittens in whom resuscitation was started with 21% compared to 100% O_2_ (11/12 vs. 1/8, *p* = 0.001). After recovering from apnea, respiratory rate was significantly lower and variability in inter-breath interval was significantly higher in kittens who received iPPV with 21% compared to 100% O_2_. FRC was not different between study groups at both timepoints.

**Conclusion:** Initiating resuscitation with 100% O_2_ resulted in increased respiratory activity and stability, thereby reducing the risk of apnea and need for iPPV after birth. Further studies in human preterm infants are mandatory to confirm the benefit of this approach in terms of oxygenation. In addition, the ability to avoid hyperoxia after initiation of resuscitation with 100% oxygen, using a titration protocol based on oxygen saturation, needs to be clarified.

## Introduction

Most preterm infants breathe spontaneously at birth, but their pulmonary gas exchange is usually insufficient due to a weak respiratory effort, low respiratory muscle strength, and lung immaturity (1–5). As a result, respiratory support is commonly needed to assist preterm infants, which mostly focuses on supporting the mechanics of breathing. For instance, continuous positive airway pressure (CPAP) is applied to support the infant's breathing efforts, or if these are absent (i.e., during apnea), intermittent positive pressure ventilation (iPPV) is used (6, 7). However, non-invasive ventilation using a face mask is often inadequate due to mask leakage or airway obstruction (1, 2). Recent animal studies have identified the larynx as the primary source of airway obstruction, which adducts during apnea and prevents air from entering the lung when iPPV is given non-invasively (8). Before birth, laryngeal adduction during apnea plays an important role in lung development as it helps to maintain the lung in an expanded state, which is the primary stimulus for lung growth (9). Nevertheless, as laryngeal adduction during apnea persists after birth (1, 2, 9, 10), spontaneous breathing will ensure that the larynx is open and air can enter the lung. This has been shown in human preterm infants as well, where applying non-invasive ventilation can result in distension of the upper airway instead of aeration of the lung (11) As such, stable spontaneous breathing is now considered essential for non-invasively applied respiratory support for very preterm infants (2, 8).

Tactile stimulation, CPAP and caffeine can all promote spontaneous breathing at birth (12–18), but as hypoxia is a potent inhibitor of spontaneous breathing, it may counter the stimulatory effect of these interventions. This would make stabilizing infants at birth difficult, particularly when the fraction of inspired oxygen (FiO_2_) is low. Historically, a set FiO_2_ of 1.0 was used to resuscitate preterm infants and oxygen saturation (SpO_2_) levels were not consistently monitored (4, 19). However, when it became clear that hyperoxia can cause tissue injury (20–22), the guidelines were revised and focused on preventing hyperoxia. These now recommend that resuscitation should start with a low FiO_2_ (0.21–0.3) and then be titrated based on the infant's SpO_2_ (23–26). As a result, longer periods of hypoxia are now tolerated after birth, but little is known about how this impacts the infant's transition to newborn life.

In very preterm infants, hypoxia at 5 min after birth is associated with a lower heart rate, a higher mortality before hospital discharge and a higher risk of intraventricular hemorrhage (27). As the inhibitory effect of hypoxia on breathing, which also causes laryngeal adduction (28), persists for weeks after birth (29), avoiding hypoxia must be an essential component of any strategy that promotes spontaneous breathing at birth. However, translating this knowledge into algorithms that define how FiO_2_ levels should be changed to adequately oxygenate newborn infants, is very complex. This is because the presence of airway liquid at birth greatly reduces the surface area for gas exchange, necessitating the use of a higher partial pressure gradient for O_2_ diffusion to achieve adequate oxygen exchange. As the surface area increases exponentially with lung aeration, the need for this higher partial pressure gradient must decrease exponentially, necessitating a rapid reduction in the FiO_2_ to avoid hyperoxia.

As partial lung aeration at birth is common in very preterm neonates, we hypothesized that using a high FiO_2_ at birth reduces the risk of a hypoxia-induced inhibition of breathing, leading to higher respiratory rates and more stable breathing. Furthermore, we hypothesized that a more stable breathing pattern will lead to better aeration of the lung (measured as functional residual capacity; FRC), leading to a rapid reduction in the need for a high FiO_2_.

## Methods

All animal procedures were approved by the SPring-8 Animal Care and Monash University's Animal Ethics Committees. The study was conducted in experimental hutch 3 of beamline 20B2 in the Biomedical Imaging Center at the SPring-8 Synchrotron in Japan.

### Experimental Procedure

Seven pregnant New Zealand White rabbits at 29 days gestation (term ~32 days) were sedated using propofol (8 mg/kg iv bolus, Rapinovet, Merck Animal Health), followed by a maintenance dose of 48–60 mg/kg/h. A 22G catheter (BD 405254) was inserted into the lower spine to administer a spinal block using 2% lignocaine (4 mg/kg) + 0.5% bupivacaine (1 mg/kg). After establishing spinal anesthesia, the propofol infusion ceased, but sedation continued with an infusion of butorphanol (0.5 mg/kg/h) and midazolam (1.0 mg/kg/h). The rabbit's reflexes (hind quarters), respiratory rate, SpO_2_ and heart rate were constantly monitored. The rabbit kittens were delivered by cesarean section, one at a time, and an esophageal tube was inserted (to measure intrathoracic pressure) and a facemask applied before the umbilical cord was cut. The kittens were weighed and naloxone (1 mg/kg) and anexate (10 μg/kg) were administered intraperitoneally to reverse the effects of maternally administered butorphanol and midazolam respectively, and a dose of caffeine base (20 mg/kg) was administered to stimulate spontaneous breathing. The kitten was placed laterally (right side down) on a heated platform positioned for synchrotron X-ray imaging. ECG leads were attached and the facemask was connected to a custom-built mechanical ventilator (30) to apply a CPAP that incorporated a bias gas flow.

All kittens were started on CPAP, commencing with a pressure of 15 cm H_2_O, which was titrated down to 8 cm H_2_O at a rate of 2 cm H_2_O/30 s. A gas blender attached to the ventilator was used to regulate the FiO_2_. Kitten heart rate (from ECG) and facemask and esophageal pressures were recorded using Labchart (Powerlab, ADInstruments, Sydney, Australia).

**Figure 1:**
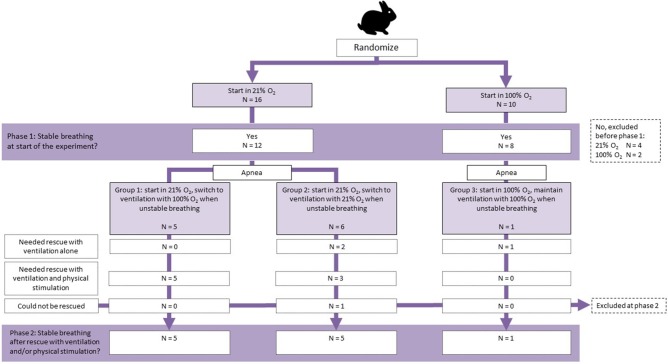
Flow diagram of allocation and outcome of kittens during phase 1 (before apnea) and phase 2 (after apnea).

The experiment commenced when the kitten was attached to the equipment and was breathing spontaneously. The experiment consisted of two phases (Figure 1). Kittens were initially randomized prior to delivery into two groups and commenced breathing in either 21% O_2_ or 100% O_2_ (phase 1), with the kittens being imaged and the breathing pattern being monitored. When spontaneous breathing ceased for >20 s, the kittens were defined as being apneic, and at that point a rescue intervention was performed using iPPV at a ventilation rate of 60 breaths per minute, with a peak inflation pressure of 25 cm H_2_O and PEEP of 8 cm H_2_O. The FiO_2_ during the rescue ventilation depended on the group. Kittens that commenced in 21% O_2_ were randomized to receive iPPV rescue with either 21% O_2_ or 100% O_2_ according to randomization, whereas kittens that commenced in 100% O_2_ remained in 100% O_2_. Whenever ventilation alone was not sufficient for regaining spontaneous breathing, physical stimulation was applied by gently rubbing the back of the kitten. When breathing was restored in such a way that the kitten remained breathing > 30 s without the need for ventilation or physical stimulation, breathing pattern was again analyzed (phase 2).

All animals were humanely euthanized at the end of the experiment using pentobarbitone sodium (>100 mg/kg) administered intravenously in the doe and intraperitoneally in the kittens.

### PC X-Ray Imaging

High resolution phase-contrast X-ray imaging was used to measure lung gas volumes via power spectrum analysis as described previously by Leong et al. (31). A Hamamatsu ORCA Flash C11440-22C detector was used with a Gadox 25 μm phosphor and a tandem lens system, giving an effective pixel size of 15.2 μm. The synchrotron radiation was tuned to 24 keV and the X-ray source-to-kitten distance was ~210 m, whereas the kitten-to-detector distance was set to 2.0 m. Imaging commenced as soon as possible after delivery, and was paused if physical stimulation of the kitten was required. The kittens were imaged with an exposure time of 20 ms and a frame rate of 10 Hz. Images acquisition was recorded in LabChart (ADInstruments, Bella Vista, NSW, Australia) to align the imaging and physiological data. Images were flat and dark field corrected and any features that may interfere with the power spectrum analysis were edited out of image and replaced with the mean pixel value of the lung region, e.g., ECG leads, the sample stage, and the edge enhanced tissue/air boundary. The power spectrum analysis and lung gas volume calibration was done on the whole lung and not lung quadrants as reported by Leong et al. (31).

A power spectrum analysis on the speckle pattern produced by overlapping alveoli in projection was used to assess lung gas volumes. The integral of the azimuthally averaged power spectrum varies in response to changing aeration of the alveoli and thus is related to the lung gas volume. The analysis was done on each frame of the image sequence, and the data points that corresponded to FRC were extracted for analysis.

### Data Analysis

During the 2 experimental phases, the stability of breathing was assessed by measuring the respiratory rate and the variability in breath-to-breath interval from intrathoracic (esophageal) pressure recordings; the variability in breath-to-breath interval was assessed by measuring the coefficient of variation (COV) of the inter-breath interval for fixed periods of time. With this analysis, a higher respiratory rate and a lower inter-breath interval COV indicates a more stable breathing pattern. In addition, heart rate was assessed in both phases of the experiment and compared between the groups. The timepoint of apnea onset, duration of apnea and amount of rescue intervention needed (none/iPPV/iPPV and physical stimulation/could not be rescued) were also compared.

As apnea occurred at different time-points after birth in different animals, the timepoint at which a stable breathing pattern was achieved following apnea was used as the start of phase 2. The average time point at which this occurred in kittens that became apneic was used as the start of phase 2 in non-apneic kittens, so that the respiratory rate and inter-breath interval COV could be compared during phase 2 in all kittens.

Phase contrast X-ray imaging was used to determine FRC during phase 1 and phase 2. In addition, the differences between the study groups with regard to FRC over time during each phase were compared.

### Statistical Analysis

Statistical analysis was performed with SPSS software version 23.0 (SPSS, Chicago, Illinois, USA). Continuous data are presented as mean ± standard deviation (SD) or median (interquartile range, IQR) depending on distribution. Variables that were assessed once in each kitten were compared using either a Student's *t*-test or one-way ANOVA when normally distributed, or a Mann-Whitney *U*-test or Kruskal-Wallis test when normality was not confirmed. A Fisher's exact test was used for categorical variables. In case of repeated measures, a linear mixed model was used to test differences between the groups, after appropriate transformation to meet normality. Tests were performed two-sided, *p*-values <0.05 were considered statistically significant.

## Results

A total of 26 kittens were randomized (Figure 1); 16 in the 21% O_2_ group and 10 in the 100% O_2_ group. Six kittens were excluded because they did not reach a stable breathing pattern that would allow the imaging and subsequent experiment to commence (Figure 1). There were no differences between randomization groups with regard to birth weight (21% O_2_-group: 34.6 ± 4.5 g vs. 100% O_2_-group: 31.8 ± 5.7 g, *p* = 0.229) or caffeine dose administered (21% O_2_-group: 18.7 ± 1.4 mg/kg vs. 100% O_2_-group: 19.2 ± 2.9 mg/kg, *p* = 0.593).

### Apnea

11/12 kittens who started breathing in 21% O_2_ became apneic and needed rescue intervention (iPPV), compared to 1/8 kittens in the 100% O_2_ group (*p* = 0.001). The time point (3.7 min) at which apnea occurred in the 1 kitten receiving 100% O_2_ was similar to the mean ± SD time of apnea onset in kittens that received 21% O_2_ (3.2 ± 1.8 min; *p* = 0.802).

Of the 11 kittens in the 21% O_2_ group that became apneic during phase 1 of the experiment, 5 were randomized to receive rescue iPPV with 100% O_2_ and 6 to rescue iPPV with 21% O_2_ (Figure 1). Rescue iPPV was started after kittens became apneic at a median (IQR) time of 22.8 (8.4–30.2) s after spontaneous breathing ceased. Additional physical stimulation was needed in 5/5 kittens who were rescued with 100% O_2_ and 4/6 kittens who were rescued with 21% O_2_. One kitten that received 21% O_2_ as part of the rescue treatment did not re-establish a sustainable breathing pattern (Table 1), and 4 kittens were only rescued for a short period of time before becoming apneic again. The duration of rescue iPPV until spontaneous breathing was re-established was not different, whether this was performed with 21% O_2_ or 100% O_2_ (206.5 ± 70.9 s vs. 186.2 ± 52.8 s, *p* = 0.299).

**Table 1:** Need for rescue therapy in case of apnea.

	**21% O_**2**_/100% O_**2**_-group** ***N* = 5**	**21% O_**2**_/21% O_**2**_-group** ***N* = 6**	**100% O_**2**_-group** ***N* = 1**	***P*-value**
**Duration of apnea (min)**^**a**^	3.0 ± 0.8	2.9 ± 0.6	2.4	0.725
**Duration of rescue therapy (s)**^**a**^	206.5 ± 70.9	186.2 ± 52.8	91.6	0.299
**Rescue therapy provided** ***n*****(%)**^**c**^				0.106
iPPV	0 (0.0%)	2 (33.3%)	1 (100.0%)	
iPPV & physical stimulation	5 (100.0%)	3 (50.0%)	0 (0%)	
Could not be rescued	0 (0.0%)	1 (16.7%)	0 (0%)	

*Data is presented as mean ± SD (a), median (IQR) (b), or n (%) (c)*.

The only kitten that became apneic after starting the experiment in 100% O_2_, also received rescue intervention with 100% O_2_. This rescue intervention started 58 s after breathing ceased, consisted solely of iPPV (no stimulation) and continued for 91.6 s before it was ceased because the kitten had regained a spontaneous breathing pattern.

### Stability of Breathing

**Figure 2:**
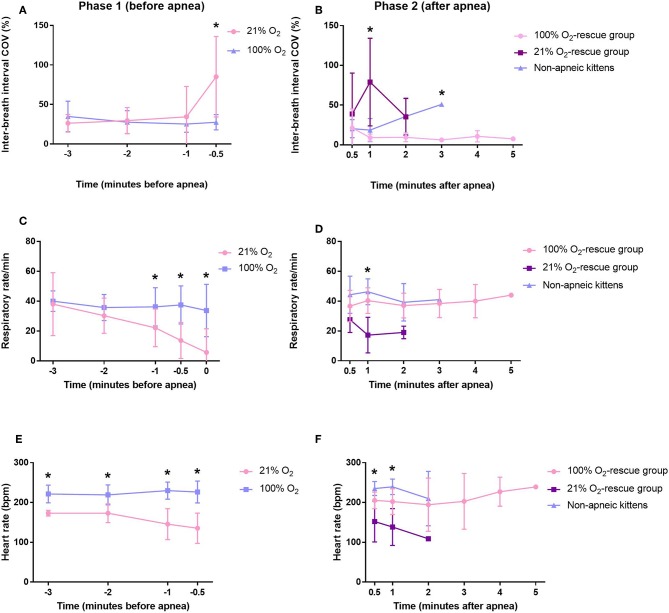
Variability in inter-breath interval and respiratory rate of rabbit kittens during the experiment. **(A)** Variability in inter-breath interval during phase 1 (before apnea). **(B)** Variability in inter-breath interval during phase 2 (after apnea). **(C)** Respiratory rate during phase 1 (before apnea). **(D)** Respiratory rate during phase 2 (after apnea). **(E)** Heart rate during phase 1 (before apnea). **(F)** Heart rate during phase 2 (after apnea). ^*^p < 0.05.

At the beginning of phase 1, both respiratory rate and inter-breath interval COV were not different between kittens who started the experiment in 21% O_2_ and 100% O_2_ (Figures 2A,C). However, toward the end of phase 1, kittens in the 21% O_2_-group had a higher inter-breath interval COV and a lower respiratory rate, which eventually resulted in apnea (Figures 2A,C). When averaged over all of phase 1, kittens who started in 21% O_2_ had a significantly higher inter-breath interval COV [33.30 (17.24–50.06)% vs. 27.54 (18.58–36.34)%, *p* = 0.009] and significantly lower respiratory rate [16 (7–28) bpm vs. 38 (32–46) bpm, *p* = 0.001] than kittens who started in 100% O_2_.

Phase 2 of this study compared the rescue of kittens from apnea using either 100% O_2_ vs. 21% O_2_ and so only included kittens that became apneic and then could be rescued. This included 11/12 kittens who commenced breathing with 21% O_2_ and 1/8 kittens who commenced breathing with 100% O_2_; only 1 kitten that commenced breathing with 21% O_2_ could not be rescued. Kittens that did not become apneic were used as a comparator. Immediately after rescue, there was no significant difference in inter-breath interval COV or respiratory rate between the groups. However, averaged over the entire phase 2 (after apnea), the inter-breath interval COV was significantly higher (*p* = 0.003) and the respiratory rate significantly lower (*p* = 0.001) in kittens in the 21% O_2_-rescue group compared to the 100% O_2_-rescue group (Figures 2B,D, Table 2). There were no significant differences in respiratory rate between the 100% O_2_-rescue group and the non-apneic kittens (Figure 2D). The inter-breath interval COV of non-apneic kittens did not significantly differ to the groups who needed rescue from apnea, until minute 3 after apnea where inter-breath interval COV was significantly higher in non-apneic kittens compared to the 100% O_2_-rescue group (*p* = 0.028; Figure 2B).

**Table 2:** Outcomes phase 2.

	**100% O_**2**_-rescue group**	**21% O_**2**_-rescue group**	**Non-apneic kittens**	***P*-value**
Variability in inter-breath interval (%)^a^	7.96 (5.84–12.94) *N* = 6	39.00 (9.95–105.92) *N* = 5	23.03 (6.69–40.34) *N* = 7	0.079
Respiratory rate (breaths/min)^a^	41 (34–44) *N* = 6	23 (15–30) *N* = 5	41 (34–52) *N* = 7	0.017
Heart rate (bpm)^a^	219 (187–236) *N* = 6	145 (102–184) *N* = 5	239 (228–253) *N* = 7	0.227
Functional residual capacity (ml/kg)^b^	23.84 ± 5.91 *N* = 5	24.25 ± 1.68 *N* = 4		0.915

*Data is presented as median (IQR), p-values are reported of linear mixed model (a), or mean ± SD, p-values are reported of linear mixed model (b)*.

### Heart Rate

During phase 1, kittens in the 21% O_2_-group had a significantly lower heart rate compared to kittens in the 100% O_2_-group (153 ± 35 bpm vs. 225 ± 23 bpm, *p* = 0.049), with heart rates being significantly reduced at all time points (Figure 2E).

During phase 2, at 30 s and 1 min after rescue, kittens in the 21% O_2_-rescue group had significantly lower heart rates compared to the other groups (Figure 2F). While this significant difference did not persist throughout the other time points within this phase they remained lower than both other groups. Averaged over the entire phase 2, there were also no differences in heart rate between groups (Table 2).

### Functional Residual Capacity (FRC)

**Figure 3:**
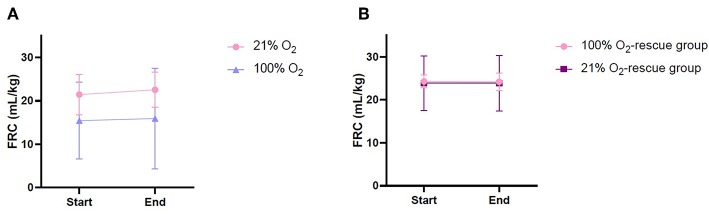
Functional residual capacity of rabbit kittens during the experiment. **(A)** Phase 1, before apnea. **(B)** Phase 2, after apnea.

FRC of kittens in both the 21% O_2_ and 100% O_2_ group showed considerable variability between individuals in phase 1, particularly in the 100% O_2_ group. There was no significant difference in FRC between groups during phase 1, although kittens in the 21% O_2_-group tended to have higher FRCs than the 100% O_2_-group (21% O_2_-group: 22.0 ± 4.3 ml/kg vs. 100% O_2_-group: 15.7 ± 10.0 m./kg, *p* = 0.894; Figure 3A). It is of interest that the kitten (1/8) in the 100% O_2_-group that became apneic, had the lowest average FRC (4.0 ml/kg) for that group over phase 1 and only had a of FRC 1.9 ml/kg just before it became apneic.

FRC levels were again measured following rescue iPPV, once the kittens had re-established a stable breathing pattern (phase 2). During phase 2, the variability in FRC between individuals was reduced and FRC levels were remarkably similar in the 21% O_2_-rescue and 100% O_2_-rescue groups (Table 2, Figure 3B).

## Discussion

We have shown that initiating respiratory support of preterm rabbit kittens with 21% O_2_ resulted in a more unstable breathing pattern (higher inter-breath interval COV), a respiratory rate that gradually reduced with time and a higher incidence of apnea compared to initiating respiratory support with 100% O_2_. Similarly, in kittens that became apneic, those who received rescue iPPV with 21% O_2_ had a higher inter-breath interval COV and a lower respiratory rate than kittens rescued with 100% O_2_. However, initiating respiratory support or rescuing from apnea with 21% O_2_ vs. 100% O_2_, did not significantly affect the degree of lung aeration as assessed by measuring FRC. These data indicate that oxygenation is a dominant factor determining the stability of breathing and the avoidance of apnea in the newborn immediately after birth. As the degree of lung aeration was similar between the two groups, despite higher respiratory rates in the 100% O_2_-group, it appears that the rate of spontaneous breathing is not the sole determinant of lung aeration at birth and must combine with other factors, such as the depth of inspiration.

It is well-established that hypoxia is a potent inhibitor of breathing movements in the fetus and this inhibitory effect of hypoxia persists well into the newborn period (32). However, the application of this knowledge has been overwhelmed by concerns of hyperoxia, which led to a change in the ILCOR 2010 recommendations on O_2_ administration during neonatal transition after birth (23). This was translated into neonatal resuscitation training programs, where iPPV became the first intervention for respiratory support and preceded an increase in the initial FiO_2_, if the newborn infant was apneic at birth (33). However, we now know that the presence of spontaneous breathing is essential for the success of non-invasive respiratory support at birth, as it ensures an open larynx, allowing the lung to be aerated (8, 11). As a result, a focus on stimulating spontaneous breathing has become a priority.

Six kittens needed to be excluded before the experiment could commence, because they could not obtain a stable breathing pattern which allowed the imaging to commence. Although there were no significant differences between the groups with regard to this finding, it is unknown if the oxygen concentration of the gas could have been involved in the achievement of a stable spontaneous breathing pattern at the start of the experiment. The fragility of the kittens complicates the application of the ECG leads, esophageal tube and face mask. This might have resulted in a difference in timepoint after birth at which individual kittens received respiratory support. Therefore, kittens who had to be excluded because they could not obtain a stable breathing pattern at the start of the experiment could have been exposed to a longer period without respiratory support, leading to an increased risk for being hypoxic, resulting in respiratory depression. However, since the number of kittens that had to be excluded before the experiment could commence did not differ significantly between the groups, this has likely not have influenced the results with regard to the rest of the experiment.

We found that, at birth, when preterm rabbit kittens breathe air (21% O_2_) they quickly develop an unstable breathing pattern and have a markedly lower breathing rate than if they breathe 100% O_2_. As partial lung aeration reduces the surface area for gas exchange, it is likely that the partial pressure of oxygen in air (~150 mmHg) was insufficient to sustain the necessary rate of oxygen uptake into the preterm kittens. As a result, they progressively became more hypoxic, resulting in an increasingly unstable breathing pattern (Figure 2A) that eventually led to apnea in 11/12 kittens. However, when an O_2_ concentration of 100% was used, the partial pressure for O_2_ (~760 mmHg) is markedly higher and greatly increases the gradient for O_2_ diffusion. As a result, the higher partial pressure gradient for O_2_ likely overcame the surface area limitation, thereby increasing oxygen uptake into the kitten. This would explain the greater breathing stability and higher respiratory rate observed in the group that started breathing 100% O_2_, where only 1 of 8 kittens became apneic. It is of interest that this kitten had the lowest FRC of the 100% O_2_ group, which decreased from 11.4 ml/kg at the start of phase 1 to 1.9 ml/kg at the end of stage 1, just before it became apneic. Clearly, the degree of lung aeration in this kitten was so low, that even breathing 100% O_2_ was insufficient to overcome the lungs gas exchange surface area limitation and adequately oxygenate the kitten.

Following apnea, kittens rescued with iPPV and 100% O_2_ had a more stable breathing pattern (lower inter-breath interval COV) and a higher respiratory rate than kittens rescued with 21% O_2_. These findings are consistent with those of van Vonderen et al., who showed a similar increase in respiratory effort in preterm infants after O_2_ was increased from 21 to 100% (34). By increasing the FiO_2_, the level of hypoxia was reduced, thereby increasing respiratory stability. These findings are consistent with the concept that hypoxia at birth leads to respiratory instability and/or suppression, which compromises lung aeration and causes the larynx to close between breaths (8). This in turn leads to persisting and worsening hypoxia that initiates a vicious cycle that opposes successful transition and necessitates escalation of interventions in the delivery room, which is known to increase the risk of adverse outcomes. This scenario is consistent with the findings of Oei et al., who showed that initiating resuscitation with a lower FiO_2_ (<0.3) resulted in a higher risk of maintaining a SpO_2_ < 80% at 5 min after birth. This in turn was associated with an increased risk of intraventricular hemorrhage, neurodevelopmental impairment or death (27, 35). Also, we have found that most kittens (82%) required physical stimulation next to iPPV as rescue therapy after they became apneic. Applying iPPV aimed to enhance aeration, and thereby oxygenation, of the kittens. Aeration can only take place in case of an open airway, including the larynx. Since it has been shown that the larynx will only open in the presence of a spontaneous breath (8), the applied iPPV will only result in aeration and improved oxygenation when the kittens takes spontaneous breaths concurrently.

We found that FRC was similar in all groups, indicating that the degree of lung aeration is not necessarily linked to the level of respiratory activity (as assessed by breathing rate) in preterm newborn kittens as we hypothesized. Indeed, during phase 1, kittens that commenced breathing in 21% O_2_ tended to have a higher FRC, with less individual variability, despite a lower breathing rate than kittens breathing 100% O_2_. While this study is limited by the difference in time after birth at which imaging could commence and FRC could be measured, as the FRC difference between the groups persisted throughout phase 1, this is unlikely to have been a factor.

We have previously demonstrated that the pressure gradients generated by inspiration are responsible for the majority (>95%) of airway liquid clearance in near term rabbit kittens (36). This results in the rapid (over 3–5 breaths) accumulation of a FRC of ~15 ml/kg in the absence of any end-expiratory pressure. As such, we predicted that increased respiratory activity would be associated with higher FRC levels, but found that in these preterm kittens a higher breathing rate did not translate into higher FRC levels. There are many possible explanations for this discrepancy. For instance, although the respiratory rate was lower the depth of inspiration may have been greater in the 21% O_2_ group. Unfortunately this could not be assessed as movement-related blurring of the image precludes lung volume measurements at end-inspiration and the esophageal pressure recording is highly position dependent. As such, the pressure amplitude can vary markedly within and between kittens. Alternately, as the glottis is mostly open during stable breathing and closes between breaths when the breathing pattern is unstable, it is possible that glottis closure in the 21% O_2_ group helps to retain a higher FRC. Whereas, in the 100% O_2_ group, the glottis remains open and so the FRC may have been reduced. Indeed, the FRC volumes we measured (~15 ml/kg) in spontaneously breathing preterm kittens are similar to what we originally measured in spontaneously breathing near-term kittens, which presumably also have open glottis'.

While it may seem contradictory to state that “rescue iPPV” was given when the kittens became apneic, while also having shown that closure of the glottis during apnea prevents iPPV from ventilating the lung, this is not the case. Rescue iPPV imposes a cyclic pressure (between 5 and 25 cm H_2_O) on the upper airways, which does not transmit down into the lower airways unless the kitten takes a breath and opens its glottis. As this spontaneous breath can occur during an inflation, the pressure support can be huge (equivalent to a CPAP of 25 cm H_2_O) and varied, leading to large uncontrolled volumes of air entering the lungs. The net result is a rapid increase in oxygenation and the re-emergence of regular spontaneous breathing, however, the presence of deep inspiratory efforts are required. Indeed, 9 of the 11 kittens that became apneic required physical stimulation to trigger a breath and initiate the “rescue process.” No kittens could be rescued in the absence of spontaneous breaths.

During this experiment, the pregnant rabbits did receive an infusion of butorphanol and midazolam. However, the dose of both agents was set based on body weight of the pregnant rabbits, resulting in equal exposure to every single rabbit kitten. There were no significant differences between the treatment groups with regard to body weight of the kittens, which makes the influence of maternal sedatives less likely. Also, kittens were randomly assigned to one of the treatment groups, resulting in equal exposure to maternal sedatives in each group. Even though the maternal sedatives could have influenced the results, it is unlikely this would have influenced the difference between the groups.

Although kittens who started breathing in 21% O_2_ initially had a lower heart rate than kittens in the 100% O_2_-group, as both were in the normal range, this difference was not considered to be biologically relevant. However, the finding that heart rates gradually decreased in the 21% O_2_ group during phase 1 is consistent with the concept that these kittens gradually became more hypoxic over time, eventually resulting in apnea. An observational study investigating SpO_2_ and heart rate changes at birth demonstrated that, using current guidelines, infants ≥ 25 weeks of gestation had values similar to the reference ranges reported by Dawson et al. (26) and Lamberska et al. (37). On the other hand, using current resuscitation guidelines, infants <25 weeks of gestation had lower SpO_2_ values and a higher incidence of bradycardia, despite concerted efforts to improve their clinical transition (37). In that study, an O_2_ titration protocol was used that commenced with a FiO_2_ of 0.3 and incorporated FiO_2_ increments of 0.1–0.2 every 30–60 s. In view of our findings, it would appear that this incremental increase in FiO_2_ was insufficient to overcome the limitation for oxygen exchange associated with a reduced gas exchange surface area. As result, the infants became progressively more hypoxic, which eventually suppressed breathing. This may have been compounded by the fact that there is a significant delay between adjusting oxygen levels at the oxygen blender and the infant receiving altered oxygen concentrations at the distal part of the circuit when using a T-piece ventilator (38). As such, it is still not clear which titrating protocols provide optimal oxygenation at birth, but the current study suggests that initiating resuscitation with a higher FiO_2_ might lead to better oxygenation. Better oxygenation may reduce the risk of a hypoxia-induced inhibition of breathing, which has the important outcome of facilitating opening of the larynx and thereby providing access to the lung for non-invasive airway pressure support.

This study is also limited by our inability to accurately measure the oxygenation level in our preterm kittens. While we administered 21% O_2_ or 100% O_2_, we do not know what PaO_2_ and SpO_2_ levels were achieved and, therefore, to what extent oxygenation of the kittens influenced the results. It is clear that hyperoxia is detrimental for preterm infants as it increases free radical production and can overwhelm their immature antioxidant capacity, causing organ damage (39–41). On the other hand, we have now shown that using too little oxygen leads to respiratory instability, a lower respiratory rate and a high risk of apnea that will necessitate a rapid escalation in the level and type of interventions required soon after birth. Thus, while the administration of 100% O_2_ has been actively discouraged for the last few years (since 2010) (23), it is timely to question whether this is appropriate. Indeed, with appropriate monitoring, the risk of hyperoxia can be minimized by titrating the FiO_2_ to keep SpO_2_ values within defined ranges, bearing in mind that the required FiO_2_ depends upon the gas exchange surface area. However, the optimal SpO_2_ range is not clearly identified in extreme preterm infants, as the internationally recommended target ranges are based on healthy term and preterm infants not requiring any resuscitation (26). Trials are underway to test the effect of initiating resuscitation with FiO_2_ of 1.0 on breathing effort, while avoiding both hypoxia and hyperoxia, as well as determining the optimal SpO_2_ target range for extreme preterm infants (42) (Clinical Trials Registry: NCT03115463).

## Conclusion

Administration of 100% O_2_ to preterm rabbit kittens at birth increased both the stability of breathing and respiratory rate when given immediately after birth or as a rescue treatment with iPPV following apnea. The results of this study provide evidence that initiating resuscitation with a high FiO_2_ might result in higher respiratory effort thereby positively influencing respiratory transition at birth. Further studies in human preterm infants are mandatory to confirm the benefit of this approach in terms of oxygenation. In addition, the ability to avoid hyperoxia after initiation of resuscitation with 100% oxygen, using a titration protocol based on oxygen saturation, needs to be clarified.

## Data Availability Statement

The datasets generated for this study are available on request to the corresponding author.

## Ethics Statement

The animal study was reviewed and approved by SPring-8 Animal Care Ethics Committee and Monash University Animal Ethics Committee.

## Author Contributions

JD, KC, MW, EM, AP, and SH made substantial contributions to conception and design of the study. JD, MC, KC, MW, EM, MK, PD, MT, TM, GR, CR, SC, AF, LC, AP, and SH performed experiments and obtained data. Data was analyzed and interpreted by JD, MC, MK, AP, and SH. The first version of the manuscript was drafted by JD, AP, and SH. All authors are acknowledged for their critical revision of the manuscript and approval of the final version.

### Conflict of Interest

The authors declare that the research was conducted in the absence of any commercial or financial relationships that could be construed as a potential conflict of interest.
